# Theory of Change: a theory-driven approach to enhance the Medical Research Council's framework for complex interventions

**DOI:** 10.1186/1745-6215-15-267

**Published:** 2014-07-05

**Authors:** Mary J De Silva, Erica Breuer, Lucy Lee, Laura Asher, Neerja Chowdhary, Crick Lund, Vikram Patel

**Affiliations:** 1 Centre for Global Mental Health, London School of Hygiene and Tropical Medicine, Keppel Street, London WC1E 7HT, UK; 2 Department of Psychiatry and Mental Health, Alan J Flisher Centre for Public Mental Health, University of Cape Town, 46 Sawkins Road, Rondebosch, 7700 Cape Town, South Africa; 3 Sangath, Alto-Porvorim, Bardez, Goa 403521, India

**Keywords:** Complex interventions, Theory of Change, MRC framework for complex interventions

## Abstract

**Background:**

The Medical Research Councils’ framework for complex interventions has been criticized for not including theory-driven approaches to evaluation. Although the framework does include broad guidance on the use of theory, it contains little practical guidance for implementers and there have been calls to develop a more comprehensive approach. A prospective, theory-driven process of intervention design and evaluation is required to develop complex healthcare interventions which are more likely to be effective, sustainable and scalable.

**Methods:**

We propose a theory-driven approach to the design and evaluation of complex interventions by adapting and integrating a programmatic design and evaluation tool, Theory of Change (ToC), into the MRC framework for complex interventions. We provide a guide to what ToC is, how to construct one, and how to integrate its use into research projects seeking to design, implement and evaluate complex interventions using the MRC framework. We test this approach by using ToC within two randomized controlled trials and one non-randomized evaluation of complex interventions.

**Results:**

Our application of ToC in three research projects has shown that ToC can strengthen key stages of the MRC framework. It can aid the development of interventions by providing a framework for enhanced stakeholder engagement and by explicitly designing an intervention that is embedded in the local context. For the feasibility and piloting stage, ToC enables the systematic identification of knowledge gaps to generate research questions that strengthen intervention design. ToC may improve the evaluation of interventions by providing a comprehensive set of indicators to evaluate all stages of the causal pathway through which an intervention achieves impact, combining evaluations of intervention effectiveness with detailed process evaluations into one theoretical framework.

**Conclusions:**

Incorporating a ToC approach into the MRC framework holds promise for improving the design and evaluation of complex interventions, thereby increasing the likelihood that the intervention will be ultimately effective, sustainable and scalable. We urge researchers developing and evaluating complex interventions to consider using this approach, to evaluate its usefulness and to build an evidence base to further refine the methodology.

**Trial registration:**

Clinical trials.gov: NCT02160249

## Background

The updated Medical Research Council (MRC) framework for complex interventions [1] is a set of guidelines for designing and evaluating complex interventions which has been widely influential in the field [2]. The framework emphasizes four phases of intervention development, feasibility and piloting, evaluation, and implementation which take place as an iterative rather than a linear process. However, the MRC framework has been criticized for not including theory-driven approaches to evaluation [3]. Although the framework does reference theory-driven approaches, it does not explicitly recommend any, or provide guidance on how to incorporate them into the design and evaluation of complex interventions [1]. The evaluation of complex interventions has also been criticized for not providing a clear explanation of the mechanisms of change through which the intervention leads to real-world impact, and for not examining how the intervention interacts with context [4]. These omissions reflect the paucity of practical examples of the use of theory-driven approaches that have been shown to work, resulting in calls for researchers to provide such examples so that the MRC framework can reflect current best practice [2,3,5].

In order to develop complex interventions which are more likely to be effective, sustainable and scalable, evaluators need to understand not just whether, but how and why an intervention has a particular effect, and which parts of a complex intervention have the greatest impact on outcomes. For this, a prospective, theory-driven process of intervention design and evaluation is required.

In this article we propose a theory-driven approach to the design and evaluation of complex interventions by adapting and integrating an existing approach, Theory of Change (ToC), into the MRC framework. We provide a guide to what ToC is, how to construct one, and how to integrate its use into research projects seeking to design, implement and evaluate complex interventions using the MRC framework.

### What is Theory of Change?

Theory-driven approaches to program evaluation can be traced back to the 1930s [6], with further development by among others Kirkpatrick in the late 1950s [7] and Chen in the 1980s [8]. Their basic tenet is that understanding the theory underlying a program approach is necessary to understand whether, and how, it works [6]. ToC developed organically, influenced by program evaluation theorists, theories of social change [9] and the work of the Aspen Institute Roundtable on Community Change in the 1990s [10-12]. This organic development has resulted in no standardized definition of ToC [13]. We will refer to ToC as that developed by the Aspen Institute and promoted by organizations such as ActKnowledge, who set up the Centre for Theory of Change and support capacity building in its use^a^.

**Figure 1:**
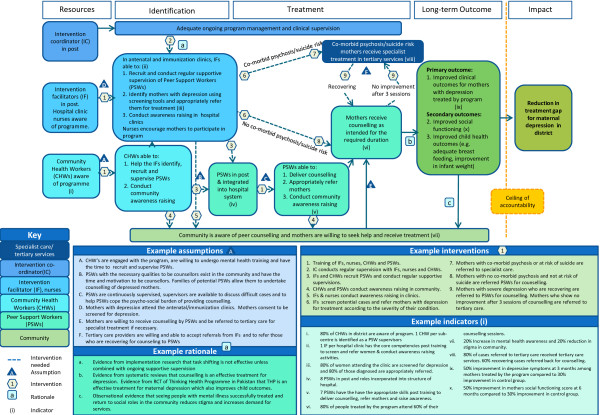
SHARE Theory of Change: peer counselling for maternal depression in Goa, India.

ToC is ‘a theory of how and why an initiative works’ [10] which can be empirically tested by measuring indicators for every expected step on the hypothesized causal pathway to impact. It is developed in collaboration with stakeholders and modified throughout the intervention development and evaluation process through an ‘ongoing process of reflection to explore change and how it happens’ [9]. It is visually represented in a ToC map which is a graphic representation of the causal pathways through which an intervention is expected to achieve its impact within the constraints of the setting in which it is implemented (see Figure 1 for an example).

ToC has been used to design and evaluate development programs in many different contexts globally [14-18]. Recognizing its capacity to provide a framework for monitoring, evaluation and learning throughout a program cycle [11], ToC is increasingly being used by international donors such as the Gates Foundation, the UK Department for International Development (DfID), Comic Relief and Grand Challenges Canada, to monitor and evaluate their research and development programs [9,13].

ToC is not a sociological or psychological theory such as Complexity Theory [19] or the Theory of Planned Behaviour [20], but a pragmatic framework which describes how the intervention affects change. The ToC can be strengthened by inserting sociological or psychological theories at key points to explain why particular links happen. For example, behavioral change theories may explain why community awareness-raising activities increase uptake of services as one link in a ToC describing how to improve maternal and child health outcomes. Equally, a ToC approach is complimentary to other frameworks which seek to reduce the chance of implementation failure, such as Normalization Process Theory (NPT) [21]. While NPT provides a framework detailing what questions should be asked to design an intervention that is more likely to be ‘normalized’ into routine practice, ToC provides an explanation for how these questions can be answered. ToC can also be used to strengthen randomized controlled trials (RCTs) and other evaluations by building and validating program theories of interventions that are then empirically tested [4].

Although similar to other theory-driven approaches to evaluation, ToC differs in a number of key ways. Logic models, for example, present a simplified model of action in a rigid linear way which articulates inputs, activities and outcomes but which does not make explicit how they are linked, or measure whether they have been achieved. Logical frameworks (log frames) are also rigidly structured and include resources, inputs, outputs, outcomes, impacts and assumptions, as well as indicators for success and specific milestones to measure. However, log frames do not necessarily explain how the various components work together in a causal pathway to achieve the impact [22], and do not link activities to outcomes. Although suitable for program monitoring and evaluation, these approaches are less useful in a research setting where the understanding of the mechanisms underlying the intervention is a key goal in unpacking the ‘black box’ of complex health interventions.

ToC has a number of advantages over these approaches. Firstly, ToC is a more flexible format which makes explicit the causal pathways through which the outcomes and activities work to achieve the desired impact, but which does not impose a pre-defined structure (such as linear structures in logic models or a cycle as in project cycle management) [23]. Instead, ToC allows for multiple causal pathways, levels of interventions and feedback loops which better reflect the reality of how complex interventions achieve their impact. Secondly, the articulation of the evidence base as the rationale for each link (pre-condition) in the causal pathway ensures that each step along the causal pathway is evidence based. Lastly, as the achievement of each pre-condition is measured through an indicator, this allows for a detailed understanding of how and whether an intervention is working and which components of a complex intervention are the most important in achieving impact.

Although ToC has been used in a research context, it is not a well-known approach in evaluation methods for complex health interventions. In a systematic review in preparation, we found 51 articles which used ToC to some extent in the design, implementation or evaluation of public health interventions (E Breuer, personal communication). However, most did not use ToC systematically throughout the research process or did not describe in significant detail how the ToC informed the development or evaluation of their intervention. None of the papers reported using ToC in RCTs or suggested using ToC together with the MRC framework.

## Methods

We are currently piloting the use of ToC to design, implement and evaluate complex interventions for mental health in a number of research projects in low- and middle-income countries. These include both RCTs and observational designs to which ToC is also suited. Throughout the paper we use the example of the South Asian Hub for Advocacy, Research and Education on mental health (SHARE) trial ^b^ to illustrate the process of developing a ToC within the MRC framework. SHARE is adapting an evidence-based counselling intervention for maternal depression delivered by Community Health Workers in Pakistan [24] to be delivered by peer support workers as this is more sustainable in a low resource context. The effectiveness of the peer-delivery system is being evaluated through a cluster RCT in Pakistan and an individual RCT in India. The SHARE example also demonstrates that ToC can be used both to develop new interventions and also to adapt existing interventions to new contexts or models of service delivery. To provide further examples, Case Study 1 describes the use of ToC in the Rehabilitation Intervention for people with Schizophrenia in Ethiopia (RISE) trial, and Case Study 2 describes the use of ToC in a non-randomized evaluation in the PRogramme for Improving Mental health care (PRIME), integrating mental health into primary care in five low- and middle-income countries.

### Ethical approval

Ethical approval for SHARE, including the ToC workshops, was granted by the Indian Council of Medical Research, Sangath Institutional Review Board, India, and the London School of Hygiene and Tropical Medicine, UK (reference 7141). Ethical approval for RISE was including the ToC workshops was granted by the Addis Ababa University College of Health Sciences Institutional Review Board (reference 039/13/PSY), the Addis Ababa University Department of Psychiatry (reference MF/PSY/212/2005) and from the London School of Hygiene and Tropical Medicine, UK (reference 6408). Ethical Approval for PRIME was granted by the University of Cape Town (reference HREC 412/2011) and from Institutional Review Boards in each of the five participating countries, as well as by the World Health Organization. Either verbal or written informed consent was obtained from all of the participants in the ToC workshops in all the projects.

## Results

The results describe how ToC was applied to each phase of the MRC framework (development, piloting, evaluation and dissemination) in the context of the SHARE trial. The two case studies provide further practical examples of how ToC can be used in combination with each stage of the MRC framework to develop and evaluate complex interventions.

### Development of complex interventions using Theory of Change

At the start of the intervention development phase, ToC uses a participatory approach by bringing together a range of stakeholders (for example health service planners, healthcare workers and service users) to develop a ToC map and to encourage stakeholder buy-in to the project [25]. This takes the form of a series of workshops, interviews or focus groups, with the choice of method based upon what is locally feasible and acceptable [15].

In the workshop, stakeholders first agree on the real-world impact they want to achieve. They then identify the causal pathways through which this change can be achieved in that context using the available resources. These are articulated as a series of preconditions leading to outcomes, the order of which can be adjusted as the pathway develops. Determining what contextual conditions are necessary to achieve the outcomes, what resources are required to implement the interventions, and how the program gains the commitment of those resources are crucial outputs of the process. There are several guidelines available which may assist with conducting ToC workshops [12,26].

Additional components of the ToC map include: identifying the interventions needed to move from one precondition on the causal pathway to the next and articulating the evidence for each link in the pathway. This rationale may be drawn from a range of sources including research evidence, behaviour change theories, local knowledge or from primary research conducted as part of the intervention feasibility and piloting stage. Drawing on a more diverse set of evidence and experience should produce a more plausible intervention. In addition, the key assumptions which set out the conditions which the causal pathway needs to achieve impact are highlighted. Through this process, potential barriers and interventions needed to overcome these barriers can be identified so that the ultimate impact can be achieved. Lastly, indicators are identified for each precondition in the pathway to evaluate whether each stage of the pathway leading to the final impact is achieved.

All these components are displayed graphically on a ToC map, often with an accompanying narrative that describes the pathways and key assumptions. Figure 1 presents the ToC map for SHARE India and Table 1 elaborates on common ToC terminology and definitions outlined above.In SHARE, the research team who developed the original intervention in Pakistan constructed a ToC map describing how the intervention worked. This was used as the basis of ToC workshops in India to modify the intervention to be delivered by peer support workers, adapt it to the Indian context and facilitate stakeholder buy-in to the project. Eighteen health professionals (9 doctors, 3 gynecologists and 2 psychiatrists) and 11 other professionals (3 counsellors, 5 staff nurses and 3 community maternal health workers) participated in a half-day ToC workshop held in the district hospital where the trial was to be conducted, facilitated by the research team. The output from the SHARE workshop comprised a ToC map (Figure 1) and a detailed report generated from an analysis of the group discussions outlining the barriers in delivering the intervention and strategies to overcome them.

**Table 1:** Common Theory of Change terminology and definitions

**Terminology**	**Definition**	**Examples**
**Impact** (ultimate outcome, goal)	The real-world change you are trying to affect. The program may contribute towards achieving this impact, and not achieve it solely on its own.	- Reduced prevalence of depression in a district.
**Longterm outcome**	The final outcome the program is able to change on its own. This will be the primary outcome of the evaluation.	- Reduced symptoms of depression in the population receiving the intervention
**Precondition** (short-term, intermediate and longterm outcomes, milestones)	The intended results of the interventions. Things that don’t exist now, but need to exist in order for the logical causal pathway not to be broken and the impact achieved.	- Staff in post to develop intervention.
		- Changes in knowledge, attitudes and skills of health workers to enable them to successfully deliver the intervention.
	The logical and sequential connections between shorter-term preconditions and longer-term outcomes that are illustrated on the ToC diagram as arrows.	
**Ceiling of accountability**	Level at which you stop using indicators to measure whether the outcomes have been achieved and therefore stop accepting responsibility for achieving those outcomes. The ceiling of accountability is often drawn between the impact and the longterm outcome.	- Project aims to change individual patient outcomes, but does not accept responsibility for changing levels of health problems in the wider population (the goal), as it cannot achieve this on its own (though it may contribute to this wider goal).
**Indicator**	Things you can measure and document to determine whether you are making progress towards, or have achieved, each outcome.	-Number of staff trained
		- Knowledge of and attitudes towards mental illness among carers
		- Percentage of people with mental illness diagnosed in primary care
		- Reduction in clinical severity of mental illness
**Interventions** (strategies)	The different components of the complex intervention.	- Training program for service providers
		- Community awareness campaign
	A dotted arrow is used to show when an intervention is needed to move from one outcome to the next.	- Inter-personal therapy
		- Antidepressant medication
	A solid arrow is used when one outcome logically leads to the next without the need for any intervention.	
**Rationale**	Key beliefs that underlie why one outcome is an outcome for the next, and why you must do certain activities to produce the desired outcome. Can be based on evidence or experience.	- Mothers and their families need to be educated about the signs and symptoms of maternal depression in order for maternal depression to be detected in the community.
**Assumptions**	An external condition beyond the control of the project that must exist for the outcome to be achieved.	- Political desire to support the program exists
		- Funder continues to fund project
		- Task-sharing is politically and culturally acceptable

### Feasibility and piloting complex interventions using Theory of Change

Before an intervention is implemented, the ToC should be tested in the feasibility and piloting phase of the MRC framework. This involves using assumptions articulated in the ToC to formulate research questions to test in formative research. This may help reduce implementation failure as weak links in the causal pathway are tested and strengthened, leading to a revision of the intervention where necessary. The ToC is then modified to reflect changes resulting from the feasibility and piloting phase and a revised ToC is taken forward for formal testing in the evaluation phase. Developing a ToC must be a continual process of reflection and adaptation as barriers to implementation arise and new evidence comes to light, requiring pathways to be changed and strengthened.The assumptions generated by SHARE’S ToC were used to generate questions to be tested in the intervention’s formative research. Key assumptions being tested through qualitative interviews with community members and mothers include ‘peer support workers with the necessary qualities to be counsellors exist in the community and have the time and motivation to be counsellors’ (Figure 1, assumption B), and ‘mothers are willing to receive counselling by peer support workers’ (Figure 1, assumption E). Other formative research methods to test key assumptions include an analysis of patient flow through the antenatal and immunization clinics where mothers with depression will be identified, an assessment of the existing referral system for specialist mental health care, and qualitative interviews with clinic staff to determine the most acceptable and feasible methods of screening mothers attending the clinics.

### Evaluating complex interventions using Theory of Change

The evaluation stage of a complex intervention using a ToC approach involves identifying at least one indicator for every precondition within that framework to measure whether it has been achieved. Indicators must be specific enough to describe what change is necessary in the precondition to move up the causal pathway (for example how many people need to be trained in order to deliver the intervention as intended). Pre-specifying the level of change needed to achieve an precondition makes it easier to design the components of the intervention to achieve that target. It also ensures that the indicators are meaningful measures of whether a precondition has been achieved or not. For example in SHARE, we measure whether the peer support workers have acquired the skills from training in order to deliver the counselling as intended, rather than simply recording how many people have been trained.

Evaluation using a ToC framework involves measuring indicators at all stages of implementation, not just an intervention’s primary and secondary outcomes. This includes a wider range of input, process, output and outcome indicators than may normally be measured, with a clear focus on measuring whether key stages in the causal pathway are achieved. ToC can therefore be used as the theoretical framework on which to base a detailed process evaluation necessary to unpack the ‘black box’ of a complex intervention [5,27]. ToC allows for multiple outcomes of the intervention to be pre-specified within a theoretical framework, thereby explicitly evaluating the multiple outcomes that complex interventions may lead to. In SHARE, multiple preconditions to be captured by the evaluation include the core competencies of peer support workers, the willingness of mothers with depression to seek and receive treatment, as well as the long-term outcomes of the impact of the intervention on maternal clinical, social and economic outcomes, as well as on child health.

As a result, an evaluation based on ToC will require a number of different methods to capture all of the indicators. In SHARE, the evaluation includes an RCT to assess the effect of peer-counselling on patient outcomes, nested studies of the fidelity of training including an assessment of the competencies achieved by peer support workers and the quality of supervision received, and collection of clinic based data to measure key preconditions in the ToC map such as the proportion of women who are referred to peer-counselling who receive treatment, and their adherence to the sessions.

The analysis of data collected using a ToC approach has the potential to combine process and effectiveness indicators into a single analysis which can help untangle whether, how and why an intervention has an impact in a particular context, and whether it may be suitable for scale-up or for adaptation to new settings. In order for this to be achieved, appropriate modelling techniques need to be applied, drawing on methods from other fields such as structural equation modelling [28], discrete-event simulation models [29], agent-based modelling [30], and system dynamics modelling [31]. The application of these methods to the analysis of complex interventions is an important area for further research.

### Implementing complex interventions using Theory of Change

Experience of implementation and evidence gathered from the evaluation is combined to revise the ToC and produce the final ‘story’ of how the intervention worked in a particular setting. This provides a comprehensive description of the intervention which can be disseminated to a variety of audiences, providing information on the components of the intervention that need to be adapted for use in other settings. The MRC guidance calls for more detailed and standardized descriptions of complex interventions in published reports to facilitate exchange of knowledge and to encourage synthesis of results from similar studies [1,32]. As the projects described in this paper are still ongoing, it remains to be tested whether ToC is a useful tool to meet this challenge. A full description of Case Study 1 and Case Study 2 can be found below.

### Case Study 1 | Use of Theory of Change in the RISE Trial

#### Background

The RISE trial (Rehabilitation Intervention for people with Schizophrenia in Ethiopia) aims to develop and test in a cluster-randomized trial, community-based rehabilitation (CBR) for people with schizophrenia in Sodo, a rural district in Ethiopia. CBR is a multi-sectoral method for improving social inclusion and functioning in people with disabilities [33]. CBR has been shown to improve outcomes in people with schizophrenia in India [34], but intervention development work was needed to design an intervention suitable for Ethiopia, a setting with fewer public sector resources. A situational analysis, literature review and review of existing CBR guidelines and projects were undertaken first. This allowed us to identify potential CBR components for RISE, including health (for example adherence support), social (for example social skills training), livelihood (for example, support returning to work), empowerment (self-help groups) and education (literacy group) elements.

#### Development of the intervention

Two ToC workshops were held with key stakeholders to determine the feasibility of delivering these intervention components in Sodo district. The first half-day workshop involved eight national experts in CBR and mental health. The second half-day workshop was held in Sodo and included 20 community leaders, including district-level representatives of microfinance, education, police, traditional healers and religious leaders. The ToC map was created at the first workshop and presented to and refined in the second workshop. Additional file 1 lists a summary version of the ToC map. Through these workshops, the CBR components were finalized and the key delivery structures were developed. For example, the key decision was made that CBR should be delivered by CBR workers, specially recruited and trained for RISE, rather than existing government community health workers. The workshops also allowed us to recognize the richness of local resources, and how these might be utilized for CBR, for example literacy groups and edirs (burial associations).

#### Feasibility and piloting of the intervention

Following the ToC workshop, we conducted 16 qualitative interviews and five focus groups with people with schizophrenia, caregivers, community leaders, existing CBR workers (for people with physical disabilities), and community and primary healthcare workers to test the assumptions identified in the ToC map. For example, a key concern was that it would be difficult to find and retain local CBR fieldworkers willing to work with people with schizophrenia, due to concerns about safety and stigma. The qualitative interviews showed that if adequate safety and supervision mechanisms were provided (for example risk assessment) recruitment and retention would be possible. A second assumption, that community leaders would be willing to participate without personal gain, generated conflicting views from different stakeholder groups. Female caregivers, based on their previous experiences, were skeptical that community leaders would provide support, whilst community leaders themselves were keen to collaborate. These differing opinions highlighted the importance of the pilot in understanding how CBR will work in practice. The ToC map was amended using the qualitative results and will continue to be adapted following the pilot, which will be conducted in mid-2014.

#### Evaluation of the intervention

The preconditions, long-term outcomes and indicators arising from the ToC map were used to plan a comprehensive and meaningful evaluation for RISE which combines an assessment of both the effectiveness of the intervention and also the process of implementation. One strength of CBR is that it is tailored to individual needs, meaning each CBR recipient receives a different ‘version’ of CBR. However, this means it is difficult to evaluate which CBR component, or synergy between components, results in positive outcomes for recipients. Using ToC allowed us to conceptualize how different CBR components fit onto the causal pathway to improved functioning in people with schizophrenia, and to develop appropriate ways to evaluate each component. Ultimately this will allow us to determine the active ingredients of CBR and how the process of implementation affects outcomes, in order to adapt and refine the intervention for scaling up in Ethiopia, or to translate it for implementation in new settings.

#### Challenges

A challenge of using ToC was the difficulty in operationalizing true ownership of the ToC map by stakeholders in the workshops. Although stakeholders provided the content, the map itself was created and ‘owned’ by the researchers throughout the process. This may have been due to the short time frame for explaining the concepts behind both ToC and CBR, before asking for participation in creating the map.

### Case study 2 | Use of Theory of Change in the PRrogramme for Improving Mental health care (PRIME)

#### Background

PRIME is developing and evaluating district level mental health care plans integrating mental health services into primary care in five low- and middle-income countries (India, South Africa, Ethiopia, Uganda and Nepal) [35]. Within PRIME, we used ToC as a conceptual framework underpinning the development and evaluation of the mental health care plans at a country level and also at a cross-country level to provide a framework highlighting commonalities across all five countries. The use of ToC in the PRIME program is described in detail elsewhere [36].

#### Development of the intervention

The PRIME Cross Country ToC was developed with 15 members of the PRIME team from all countries at a workshop in Goa, India at the start of the program. This initial ToC described the causal pathways of how the PRIME interventions would need to work in order to achieve the ultimate impact of ‘improved health, social, and economic outcomes for people with priority disorders and their families/carers in the PRIME districts’. A summary version of the PRIME Cross Country ToC is shown in Additional file 2.

Following the drafting of the cross-country ToC, individual countries developed district specific ToCs during a series of ToC workshops which are described in detail elsewhere [36]. In brief, between two and four workshops were held in each country with stakeholders including policymakers, district level health planners and management, mental health specialists, researchers, and service providers. The size of the workshops varied significantly between countries with a median of 15 (interquartile range 13 to 22) stakeholders attending each workshop. The workshops provided an opportunity to develop logical, evidence-based ToC maps with stakeholders, contextualize the mental health care plans and elicit buy-in from stakeholders and acted as a forum for knowledge exchange between researchers and stakeholders. Stakeholders provided detailed knowledge on the functioning of the health system and information about local resources which could be mobilized for the implementation of the mental health care plans. The researchers provided guidance on the development on the ToC, the evidence available for potential interventions, as well as strategies to evaluate the success of the plans. The ToC maps were further developed after the ToC workshops and used as a basis for the development of the district specific mental health care plans, in combination with a variety of other methods including a situational analysis [37], a costing tool, and interviews and focus group discussions with key stakeholders. The Cross Country ToC was then further refined by comparing it to the district specific ToC maps to ensure that all the key preconditions and long-term outcomes across countries were captured.

#### Feasibility and piloting of the intervention

The cross-country ToC highlighted a number of assumptions which were used to develop cross-country topic guides for formative semi-structured interview guides and focus group discussions with stakeholders designed to test the feasibility of the interventions. These were supplemented by questions designed to answer country-specific assumptions taken from the district level ToCs. The subsequent qualitative interviews and focus groups gathered information in each country on access and demand for mental health care, service delivery recovery and rehabilitation and accountability. The results of this formative research were used to refine the district specific ToCs and develop the mental health care plans in each country.

#### Evaluation of the intervention

The indicators for the cross-country ToC were refined using the indicators from the district specific ToC maps and compared across countries to identify common indicators across countries that could be used as the basis of an evaluation strategy to answer cross-country research questions such as whether the mental health care plans reduce the treatment gap in the districts, and whether the patients treated by the programs have improved clinical, social and economic functioning. These indicators were used to plan the evaluation design for PRIME. A variety of evaluation methodologies are being used, including detailed process evaluations, repeat cross-sectional surveys, cohort studies and RCTs. As the PRIME evaluation is ongoing, we have not yet been able to test whether the process and outcome indicators from the ToC can be combined in a single analysis or to test the usefulness of ToC in the implementation of the interventions at scale. These will be the subject of future research by PRIME.

#### Challenges

One of the challenges in PRIME was using multiple ToC maps at different levels. The PRIME cross-country ToC map provided us with an overall framework of the causal pathways required for the integration of mental health care into primary health care but did not specify the country specific context and resources. In particular, the interventions which will be implemented in each country as part of the mental health care plan are different for each district according to local feasibility, existing financial and human resources and cultural acceptability. For this reason, a locally adapted district level ToC was essential for each country to ensure that these factors are accounted for in the design and evaluation of their mental health care plan. However, having an overarching ToC allowed a cross-country view of how the programs were likely to work in all countries which led to the development of an evaluation design which could be used across all countries. Another limitation of the ToC approach is that if it is to be developed with stakeholders, it requires a significant amount of work facilitating the ToC workshops and compiling the resulting ToC. However, as this process is structured around the components of the ToC and has a defined output, it is an efficient way to conduct discussions with stakeholders [36]. Critical to the success of ToC in PRIME has been having a ‘ToC champion’ who took responsibility for coordinating with countries to help them develop their district level ToC, and drove forward the development and refinement of the cross-country ToC.

## Discussion

**Figure 2:**
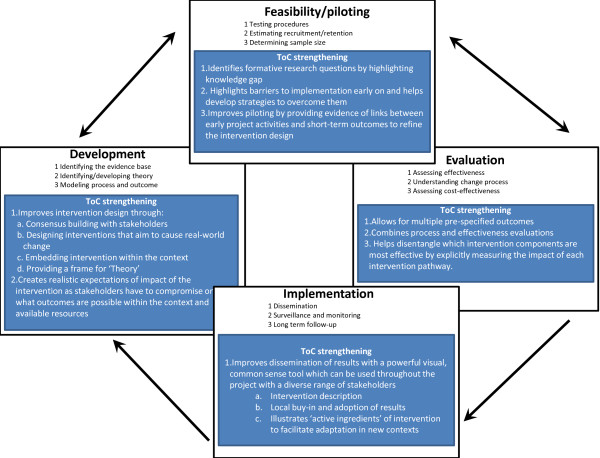
**How Theory of Change can be used to strengthen the MRC framework.** Adapted from Craig *et al.*[1].

Our experience of using ToC in three projects designing and evaluating complex interventions to improve mental health has demonstrated a number of benefits, which we believe strengthen the existing MRC framework. Figure 2 summarizes how using ToC has the potential to strengthen each phase in the MRC framework.

Using a ToC approach for the development of an intervention may enhance the MRC framework in two key ways. Firstly, using a ToC approach provides a useful framework to guide stakeholder engagement. While stakeholder participation is an increasingly an important part of health services research^c^, using a ToC approach may prompt a deeper level of engagement than other methods as it enables stakeholders to take part in the initial design of the intervention in a formal and participatory way. This was certainly true in the PRIME and RISE projects where we found a deeper level of stakeholder engagement from a relatively short workshop. However, our experience from all three projects indicates that this stakeholder engagement in the ToC process does not extend beyond the workshops, and that a ToC champion within the project is needed to drive the process forward.

Secondly, it may improve the initial design and potential effectiveness of the intervention by explicitly designing interventions which are embedded in the local context and seek to have an impact in the real world as opposed to in a research setting. Designing a feasible intervention that is likely to work in the constraints of the context and available resources is challenging. Agreeing on how interventions lead to outcomes can be politically charged if achieving those outcomes implies a major resource reallocation, or changes in work patterns away from the current status. One of the strengths of ToC is that design and implementation issues are brought centre-stage from the start, and if any aspects of the intervention are politically unacceptable, or if the resources will not be available, then all stakeholders have to compromise and come to alternative solutions to ensure that the impact is achieved. This was demonstrated in the RISE trial where very early on in the workshop it became clear that using government community health workers to deliver the intervention as we had planned would not be politically acceptable, leading the group to decide to train dedicated CBR workers.

Another advantage of the ToC process is embedding the intervention within the context in which it is to be implemented, which enables contextual factors which may affect implementation to be highlighted and tracked, along with potential unintended consequences of the intervention. This was also shown in RISE where the workshops highlighted the richness of local resources that could be utilized for the CBR intervention, such as local literacy groups and burial associations that the CBR workers could refer people to. In PRIME, we have designed district, primary healthcare facility and community level case studies to track changes in the local context (such as changes in local health priorities or staffing levels in primary healthcare facilities) that may affect the impact that the mental health care plans have. By forcing us to measure not only the process of implementing the interventions but also the context in which it is implemented, we hope to be able to conduct a much richer analysis of how and why the PRIME mental health care plans achieve any impact. This is particularly important in evaluations of complex interventions where the context may facilitate or impede the success of the intervention [38].

One key advantage of using ToC to pilot the feasibility of interventions is that it enables the systematic identification of knowledge gaps to generate research questions for the pilot stage. Completing the rationale for each link in the causal pathway highlights which linkages lack evidence and therefore what additional work is needed to fill those gaps. Secondly, highlighting specific barriers to intervention delivery early on enables strategies to overcome these barriers to be incorporated into the intervention design. An example of this from SHARE is the need for consensus building workshops with policymakers and hospital staff to change attitudes towards using peer-counsellors for treating maternal depression, which we have now made part of the intervention.

A key intended benefit of using a ToC framework for the evaluation of complex interventions, particularly in trials, is that it breaks down the barriers between evaluations of intervention effectiveness and process evaluations by combining them into one framework. Though detailed process evaluations are becoming more widely used in trials, they are rarely combined with an assessment of intervention effectiveness in a single analysis, enabling interpretation of the outcome data in light of the process data. As the three projects we describe in the paper have not yet reached the analysis stage, it remains unknown whether this benefit will be realized. Future work needs to explore ways of modelling the pathways to impact by combining process and outcome data, enabling a more nuanced assessment of which components of the intervention may be most critical for achieving the desired outcome.

Our research has shown that ToC is useful in the implementation phase of the MRC framework as it helps to develop locally adapted, contextually relevant plans developed with stakeholders, including local policymakers, which are therefore more likely to be feasible and acceptable and work within existing resource constraints. ToC may confer important benefits for the dissemination of information about an intervention as the ToC map may be a powerful visual tool for describing the key components of an intervention and how it impacts on outcomes. This can be used by other researchers to understand how the intervention worked (for example in systematic reviews [39,40]) and also be used to advocate with policymakers to facilitate the scale-up of successful interventions. Using ToC in this way will be the subject of future research in the projects described in this paper.

As with any approach, there are limitations. The lack of a standardized definition causes confusion and we urge researchers to adopt the definition used by the Aspen Institute [11]. In addition, comprehensive ToC maps may contain a lot of detail with many smaller process preconditions required to achieve impact. Using a detailed ToC with many preconditions and indicators to measure whether that precondition has been achieved may result in an exhaustive list of indicators to measure and a subsequently complex and expensive evaluation plan. This was the case in PRIME, where the demands of conducting a complex evaluation across five countries had to be balanced against the resources required to carry out such an evaluation. As a result, we had to refine the cross-country ToC map to ensure that it contained only the key preconditions and long-term outcomes necessary for the impact to be achieved, and that we only evaluated the key steps in the pathway.

Many of the benefits of the ToC approach derive from the participatory nature of the development of the ToC. If stakeholders are not sufficiently consulted or engaged in the development of the ToC, it is likely that using a ToC becomes yet another box to tick rather than a deeper exploration of the pathways to achieve impact [13]. This may particularly be the case where the decision to develop a ToC is made by the funder rather than seen as an integral part of program development, as shown by the use of ToC as part of the evaluation of the Health Action Zones in the UK. However, more than three quarters of the initiatives did not develop a ToC map as implementers felt that the development of a ToC was taking resources away from implementation [18]. In our experience, having a nominated ToC champion on the research team who is tasked with overseeing the ToC process and driving it forward throughout the project, is critical to the success of the approach.

Our experiences resonate with other examples of applications of theory-driven evaluation approaches, including ToC, which are reported in the literature. Afifi *et al.*[41] found that using a participatory approach to developing a logic model as the basis for a mental health promotion intervention for youth in a refugee community in Beirut improved the design of their intervention. In their program, a community youth committee was involved in the development of the logic model and provided input into the content and delivery format of the intervention resulting in a more relevant, feasible and sustainable intervention. Similarly, Hernandez and Hodges [42] used ToC developed with stakeholders to organize services for youth in contact with the juvenile justice system. They found that ToC assisted with creating a shared vision among stakeholders which promoted service integration across a variety of sectors. This also allowed planners to envisage what is expected within a community and how the actions of stakeholders can bring this about. Other experiences also highlight that ToC can assist with structuring and prioritizing the evaluation of complex interventions [17,43-46]. However, few provide enough detail to understand how ToC informed both the design of the program and the subsequent evaluation.

It is still in the early stages. While we have tested the use of ToC in three research projects across six countries, these are all mental health programs in low- and middle-income countries, and none have completed the evaluation, analysis or dissemination of the evaluation. Further research is needed in other settings, for other types of complex interventions, and into the usefulness of ToC as a framework for analysis and dissemination of results.

## Conclusions

This paper is the first to describe the use of ToC in conjunction with the MRC framework for the development and evaluation of complex interventions, and to provide three case studies testing this approach. Indications from our initial experiences are that, used in conjunction with the MRC framework, ToC may be a useful tool to improve the development and evaluation design of complex interventions in research projects. We urge researchers to consider using this approach and to evaluate its usefulness within a research context.

## Endnotes

^a^http://www.theoryofchange.org/

^b^http://www.centreforglobalmentalhealth.org/projects-research/share-south-asian-hub-advocacy-research-and-education-mental-health

^c^See for example: Patient and Public Involvement http://www.ccf.nihr.ac.uk/PPI/Pages/default.aspx and the James Lind Alliance http://www.lindalliance.org/.

## Abbreviations

ToC: Theory of Change; MRC: Medical Research Council; DfID: Department for International Development.

## Competing interests

The authors declare that they have no competing interests.

## Authors’ contributions

MDS conceived the idea for this project and developed the initial use of ToC for the design and evaluation of complex interventions in conjunction with the MRC framework, with support from LL, EB, CL and VP. EB, MDS, CL and VP developed the use of ToC in the PRIME program. NC, VP and MDS developed the use of ToC in the SHARE trial. LA and MDS developed the use of ToC in the RISE trial. MDS wrote the first draft of the paper. LA wrote Case Study 1 and EB Case Study 2. All authors revised and gave final approval to the paper.

## Supplementary Material

Additional file 1 Summary Theory of Change from the RISE trial. Click here for file

Additional file 2 Summary Theory of Change from the PRogramme for Improving Mental health carE (PRIME). Click here for file
