# Supplementary material for: Theory of Change: a theory-driven approach to enhance the Medical Research Council's framework for complex interventions
Source: Trials. 2014 Jul 5;15:267. doi: 10.1186/1745-6215-15-267 (PMC4227087; doi:10.1186/1745-6215-15-267)
Supplement: Additional file 1 — Summary Theory of Change from the RISE trial. [file 1745-6215-15-267-S1.pptx]

## Slide 1
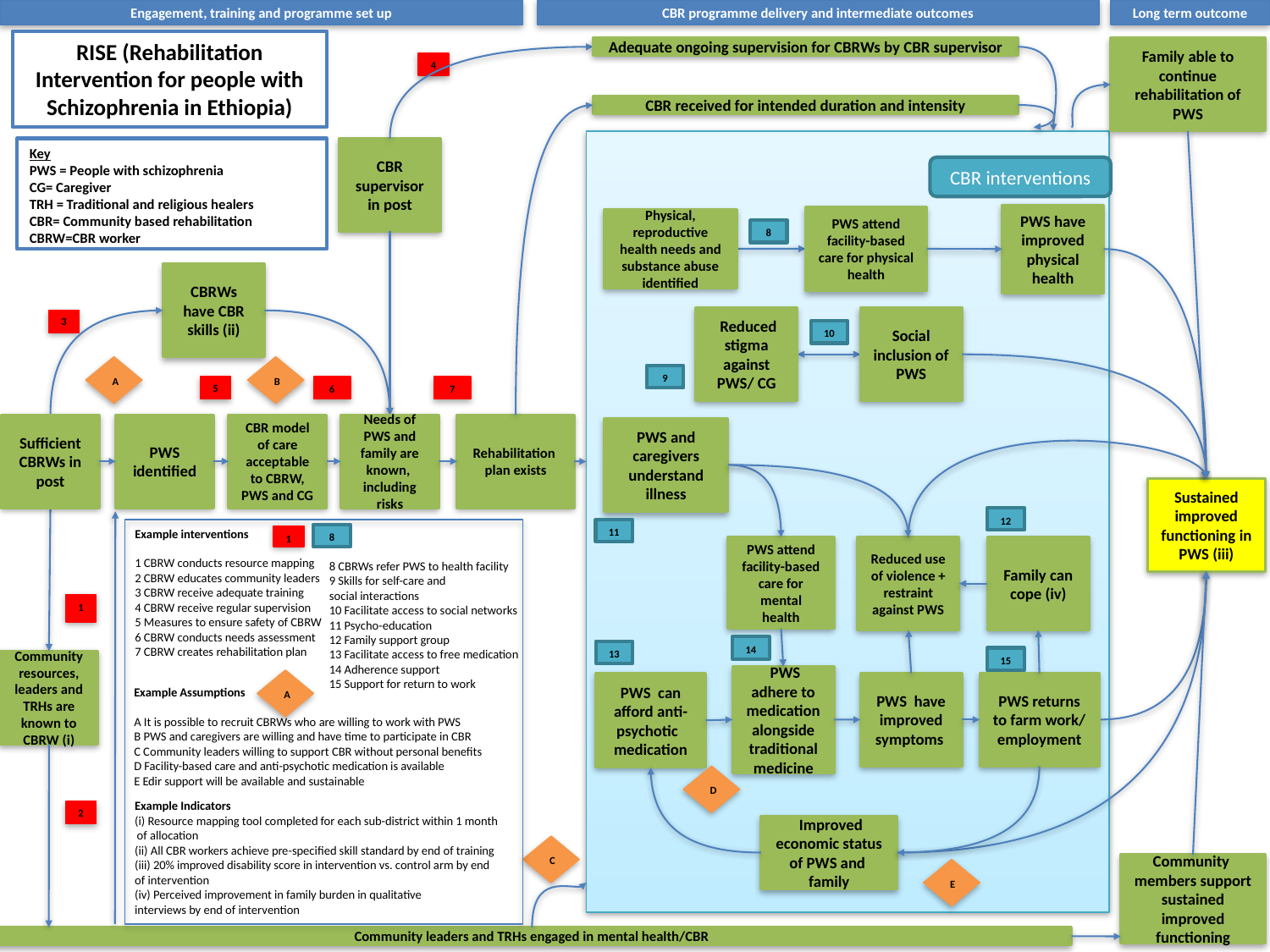

Engagement, training and programme set up
CBR programme delivery and intermediate outcomes
Long term outcome
RISE (Rehabilitation Intervention for people with Schizophrenia in Ethiopia)
Adequate ongoing supervision for CBRWs by CBR supervisor
Family able to continue rehabilitation of PWS
4
CBR received for intended duration and intensity
CBR supervisor in post
Key
PWS = People with schizophrenia
CG= Caregiver
TRH = Traditional and religious healers
CBR= Community based rehabilitation
CBRW=CBR worker
CBR interventions
PWS have improved physical health
PWS attend facility-based care for physical health
Physical, reproductive health needs and substance abuse identified
8
CBRWs have CBR skills (ii)
 Reduced stigma against PWS/ CG
Social inclusion of PWS
3
10
A
B
9
5
6
7
Sufficient CBRWs in post
PWS identified
CBR model of care acceptable to CBRW, PWS and CG
Needs of PWS and family are known, including risks
Rehabilitation plan exists
PWS and caregivers understand illness
Sustained improved functioning in PWS (iii)
12
Example interventions
1 CBRW conducts resource mapping
2 CBRW educates community leaders
3 CBRW receive adequate training
4 CBRW receive regular supervision
5 Measures to ensure safety of CBRW
6 CBRW conducts needs assessment
7 CBRW creates rehabilitation plan
11
8
1
PWS attend facility-based care for mental health
Reduced use of violence + restraint against PWS
8 CBRWs refer PWS to health facility
9 Skills for self-care and
social interactions
10 Facilitate access to social networks
11 Psycho-education
12 Family support group
13 Facilitate access to free medication
14 Adherence support
15 Support for return to work
Family can cope (iv)
1
14
13
15
Community resources, leaders and TRHs are known to CBRW (i)
Example Assumptions
A It is possible to recruit CBRWs who are willing to work with PWS
B PWS and caregivers are willing and have time to participate in CBR
C Community leaders willing to support CBR without personal benefits
D Facility-based care and anti-psychotic medication is available
E Edir support will be available and sustainable
 PWS adhere to medication alongside traditional medicine
A
PWS have improved symptoms
PWS returns to farm work/ employment
PWS can afford anti-psychotic medication
D
Example Indicators
 Resource mapping tool completed for each sub-district within 1 month of allocation
(ii) All CBR workers achieve pre-specified skill standard by end of training
(iii) 20% improved disability score in intervention vs. control arm by end of intervention
(iv) Perceived improvement in family burden in qualitative
interviews by end of intervention
2
 Improved economic status of PWS and family
C
Community members support sustained improved functioning
E
Community leaders and TRHs engaged in mental health/CBR
