# Supplementary material for: Theory of Change: a theory-driven approach to enhance the Medical Research Council's framework for complex interventions
Source: Trials. 2014 Jul 5;15:267. doi: 10.1186/1745-6215-15-267 (PMC4227087; doi:10.1186/1745-6215-15-267)

### Web Appendix B: Summary Theory of change from the PRogramme for Improving Mental health care (PRIME)

Reproduced from Breuer, E et al., *Using workshops to develop Theories of Change in five low and middle income countries: lessons from the Programme for Improving Mental Health Care (PRIME).* International Journal of Mental Health Systems (in press).


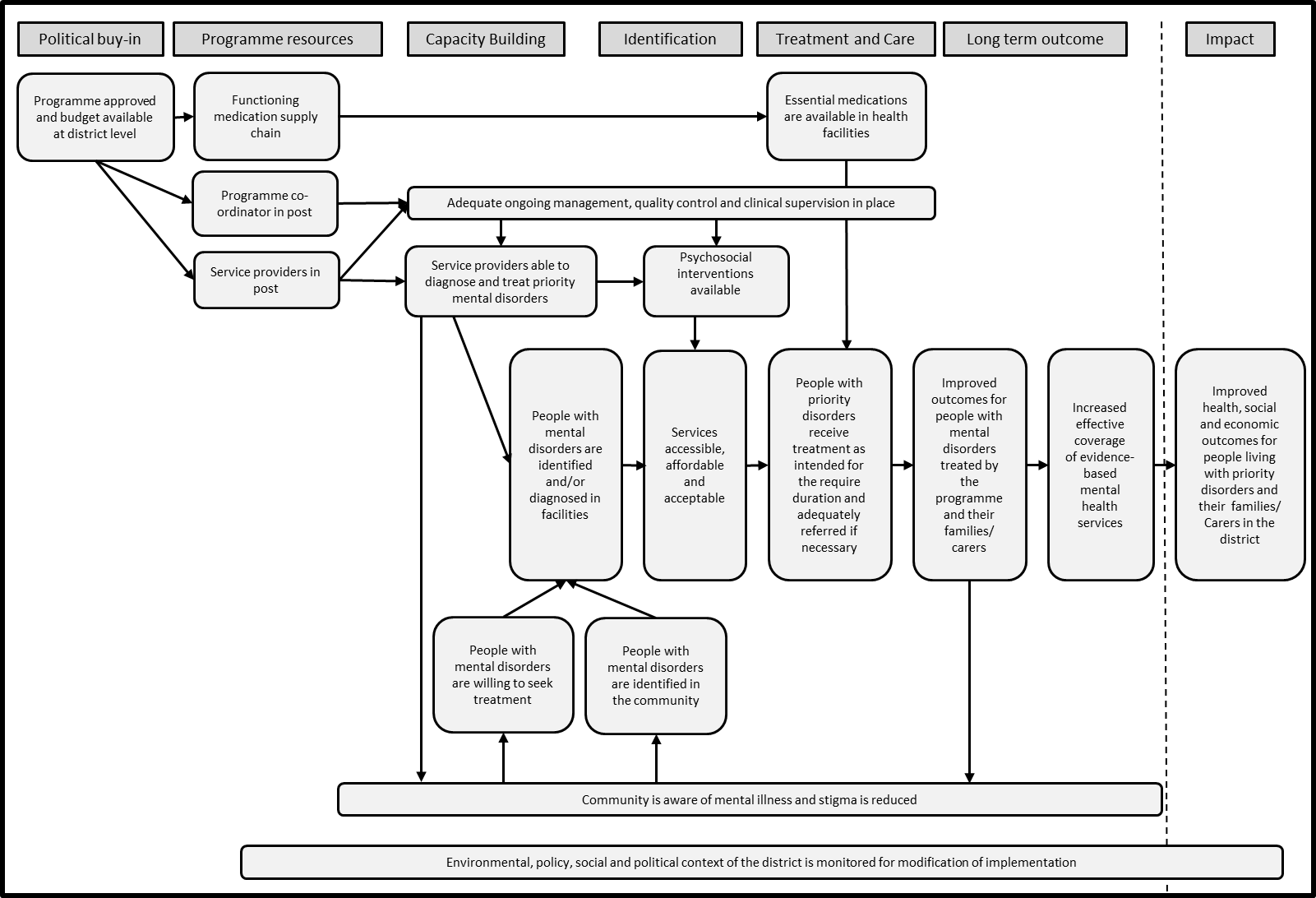

Supplement: Additional file 2 — Summary Theory of Change from the PRogramme for Improving Mental health carE (PRIME). [file 1745-6215-15-267-S2.docx]
